# Stratification-induced reorientation of disk settling through ambient density transition

**DOI:** 10.1038/s41598-017-18654-7

**Published:** 2018-01-11

**Authors:** Magdalena M. Mrokowska

**Affiliations:** Institute of Geophysics, Polish Academy of Sciences, Ks. Janusza 64, 01-452 Warsaw, Poland

## Abstract

Settling due to gravity force is a basic transport mechanism of solid particles in fluids in the Earth. A large portion of particles occurring in nature and used in technical applications are non-spherical. Settling of particles is usually studied in homogeneous ambient conditions, however, stratification is inherent of natural fluids. It has been acknowledged that stratification modifies the velocity of settling spheres and amorphous aggregates. However, the effect of particle shape on the dynamics of settling through density-stratified ambient fluid has not been recognized well enough. Here I show experimental evidence that continuous density transition markedly modifies the settling dynamics of a disk in terms of settling velocity and orientation of a particle. Settling dynamics of a disk are more complex than dynamics of spheres and aggregates studied previously. I found that in a two-layer ambient with density transition, a disk settling in a low Reynolds number regime undergoes five phases of settling with the orientation varying from horizontal to vertical, and it may achieve two local minimum settling velocities in the density transition layer. Moreover, I found that the settling dynamics depends on a density difference between upper and lower homogeneous layers, stratification strength and thickness of density transition.

## Introduction

The ocean and lakes are inhomogeneous mediums where dissolved substances and temperature act as density-stratifying agents, and nontrivial amounts of microparticles move downwards due to the gravitational force. Microparticles in the hydro-environment have various origins and structures, e.g., plankton, particulate organic matter such as zooplankton faecal pellets, marine snow, phytodetritius^1^, and microplastics^2^. Organic particles constitute a significant portion of carbon transport from the surface of the water to the depth of the ocean as part of a “biological pump”^3^ and have a significant impact on climate and ocean productivity^4^. On the other hand, microplastics found in large quantities in water, on the seafloor, and inside organisms are harmful for the environment^2^.

The fate of microparticles within a body of water depends on various factors, one of which is the density of ambient liquid and the characteristics of density stratification. In a number of water bodies, density depends on the amount of dissolved salt. Salinity of the ocean is reported to be around 3.5%, which results in a typical density of seawater of about 1.02–1.03 g cm^−3^ ^5,6^. However, salinity of natural waters such as hypersaline lakes may be much higher^7^, e.g. salinity of the Dead Sea was reported^8^ to reach 28% with a mean density of 1.23 g cm^−3^.

As they move through the water column, particles may encounter density gradients forming density transitions or density interfaces (pycnoclines)^5^. The strength of stratification is usually characterized by buoyancy frequency, *N*, with *N* up to 0.02 s^−1^ characteristic for the ocean and seas^9,10^. Higher buoyancy frequency was reported for fiords^11–13^ with the maximum *N* ranging from 0.1 s^−1^ to 0.3 s^−1^ and hypersaline lakes, e.g. *N* up to 0.12 s^−1^ for Mono Lake, California^14^ and *N* ~ 0.17 s^−1^ for the Dead Sea^15^. Sharper density gradients may be expected in sinkholes formed by a two-layer water column of freshwater and seawater^16^. While pycnoclines occurring in the ocean may reach a thickness of tens of kilometres^9^, pycnoclines in sinkholes are usually only a few meters thick^16^, which promotes a sharper density gradient.

Microparticles usually have small excess densities relative to seawater, which combined with small dimensions of particles (up to a few millimetres) results in low settling velocities^5,6^. Consequently, the particles settle in a low Reynolds number regime (Re = *du*/ν where *d* – particle diameter (m), *u* – settling velocity (m s^−1^), *ν* – kinematic viscosity (m^2^ s^−1^)), indicating a significant effect of viscous forces on particle settling. Moreover, it has been demonstrated that oceanic stratification is strong enough to affect the dynamics of small particles^17^. According to these findings, a particle larger than length scale *O*(100 μm–1 mm) experiences an impact of stratification due to the combined effect of buoyancy, diffusion and viscosity.

While density profiles in the ocean exhibit complicated linear and non-linear patterns^9,10^, numerical and experimental studies on settling particles motivated by natural conditions have been performed for rather idealized conditions, such as sharp density transitions^18–21^ and linear stratification^22–24^. In the light of the length and stratification scales in water bodies, some laboratory conditions may seem not to apply to those occurring in nature. However, the studies have demonstrated complex physical processes necessary to understand the settling of particles and its environmental implications.

It is well acknowledged that stratification suppresses the motion of particles through the vertical gradient of density. The settling velocity is smaller in the presence of stratification compared to the same density in homogeneous fluid^19,23,25^, which implies that additional stratification-induced drag is present. The impact of stratification on settling particles is characterised by the Froude number (Fr = *u*/*Nd*) expressing the relative effects of inertial and buoyancy forcers^19,23^ or by the Richardson number Ri = Re/Fr^2^ expressing the ratio between buoyancy and viscous shear forces^22^. While Ri is appropriate for a low Re number (<1) where inertia is negligible, the Fr number is applied when inertial forces are more significant^26,27^. It has been demonstrated that in linearly stratified fluid drag increases with Fr^−1^ or Ri, i.e., with increasing stratification strength^22,23,28^. This is explained by the way isopycnals are disturbed by the settling particle - they are compressed and pulled downward by the particle and then restored to their position of neutral buoyancy. The stronger stratification, the easier the isopycnals restore, imposing a higher drag on the particle^28^.

Another significant physical process affecting the settling dynamics is the diffusion of a stratifying agent^22,28^. The ratio between momentum diffusivity and the diffusivity of stratifying agent is characterised by the Prandtl number Pr = ν/κ, where κ – diffusivity of stratifying agent (m^2^ s^−1^), for salt κ = 1.5 10^−9^ m^2^ s^−1^. Typical values are Pr = 7 for temperature and Pr = 700 for salt^22^. Generally, stratification due to salinity affects the settling of particles to a higher extent since smaller diffusivity enhances the compression of isopycnals^22^.

When particle settles in stratified ambient, a lighter fluid from upper layers may be dragged by the particle to lower denser layers. Consequently, the velocity of settling particle is reduced due to the increase in buoyancy imposed by lighter fluid attached to the particle. The effect is significant in both sharp interfaces^18,19^ and in linear stratification^22^. Moreover, in sharp interface even reverse motion is possible when the density of particle is close to the density of lower layer^18^. It was shown that only a boundary layer around a particle formed by lighter fluid is responsible for velocity reduction^22^, contrary to previous notion that the entire wake of entrained fluid contributes to the drag^19^. The deceleration effect is exceptionally pronounced when the density of particle approaches the density of lower layer, because the particle settles in lower velocity and the mixing of entrained fluid with denser ambient is slower, while the light boundary layer persists for longer^18^.

Additional drag may also be the effect of internal waves. They may be induced by the settling particle itself, or by the tail of lighter entrained fluid separated from the particle and returning to its neutral buoyancy position^19,22^. In the latter case, internal waves do not contribute to drag. To generate internal waves, high inertia of settling particle is necessary, which occurs only for moderate or high Re^24,29^.

It should be stressed that the mechanisms affecting the settling of particles in the presence of ambient stratification have been studied mainly for spherical particles, while microparticles present in natural waters have various shapes, usually far from spherical: microorganisms have various irregular and regular shapes^30^, aggregates of organic matter are considered amorphous porous particles^31^, and microplastics are found in the form of plates, disks, cylinders, ect.^2,32^. Although non-spherical particles are ubiquitous not only in nature but also in engineering and technology applications^33^, to date it is unclear how properties of ambient fluid affect their settling, and Stokes law dedicated to a sphere settling in homogeneous quiescent fluid is usually applied in the low Re number regime^3,6,34^. In fact, the motion of non-spherical particles is much more complicated than the settling of spheres. Even in homogeneous fluid for specific conditions they may change orientation during settling, which modifies pressure distribution around the particle, and consequently drag^35,36^. However, the settling of non-spherical particles in background stratification is practically unstudied. Only recently, it has been demonstrated numerically that in stratified fluid, buoyancy-induced torques acting on an elongated particle affect the settling orientation and settling velocity of the particle^21^. The settling behaviour revealed in that study was a motivation for the experimental investigation presented in this paper.

Here I study the behaviour (orientation and settling velocity) of individual thin disks settling through a two-layer water column with density transition due to the vertical variation of salinity. This study is motivated by a scarce knowledge on settling dynamics in natural waters, however, presents a fundamental physical problem. Density transition was formed by a continuous non-linear stratification. Experimental conditions are different from conditions encountered in nature in terms of transition length scales and stratification strength. However, the results provide insight into physical processes that may occur in stratified environment and are crucial to our understanding of settling processes in natural waters. The findings presented in this study may be of significance to all disciplines where the settling of particles in various stratification conditions is considered - earth sciences^37–39^, ecology^40^, medical applications^41^, technology and engineering^42^.

This study is the first one showing the complex behavior of disk settling in a two-layer set up with continuous density transition. Previously, the impact of density transition on particle settling dynamics was considered for smaller transition thickness to particle diameter ratio, e.g., in numerical studies for spherical particles with the ratio ranging from 0.1 to 4.8^43^ and for elongated particles with transition thickness on the order of particle dimensions^21^. In both cases, a negligible effect of transition thickness was observed, which was considered “somewhat suprising”^43^.

I demonstrate that disks settling at low Re number undergo a stratification-induced pattern of reorientations and settling velocity variations. I positively verify the hypothesis that the characteristics of settling dynamics are affected by a density jump, i.e., the density difference between upper and lower homogeneous layers, the thickness of density transition, and the strength of stratification defined by buoyancy frequency.

## Results and Discussion

### General experimental conditions

**Figure 1:**
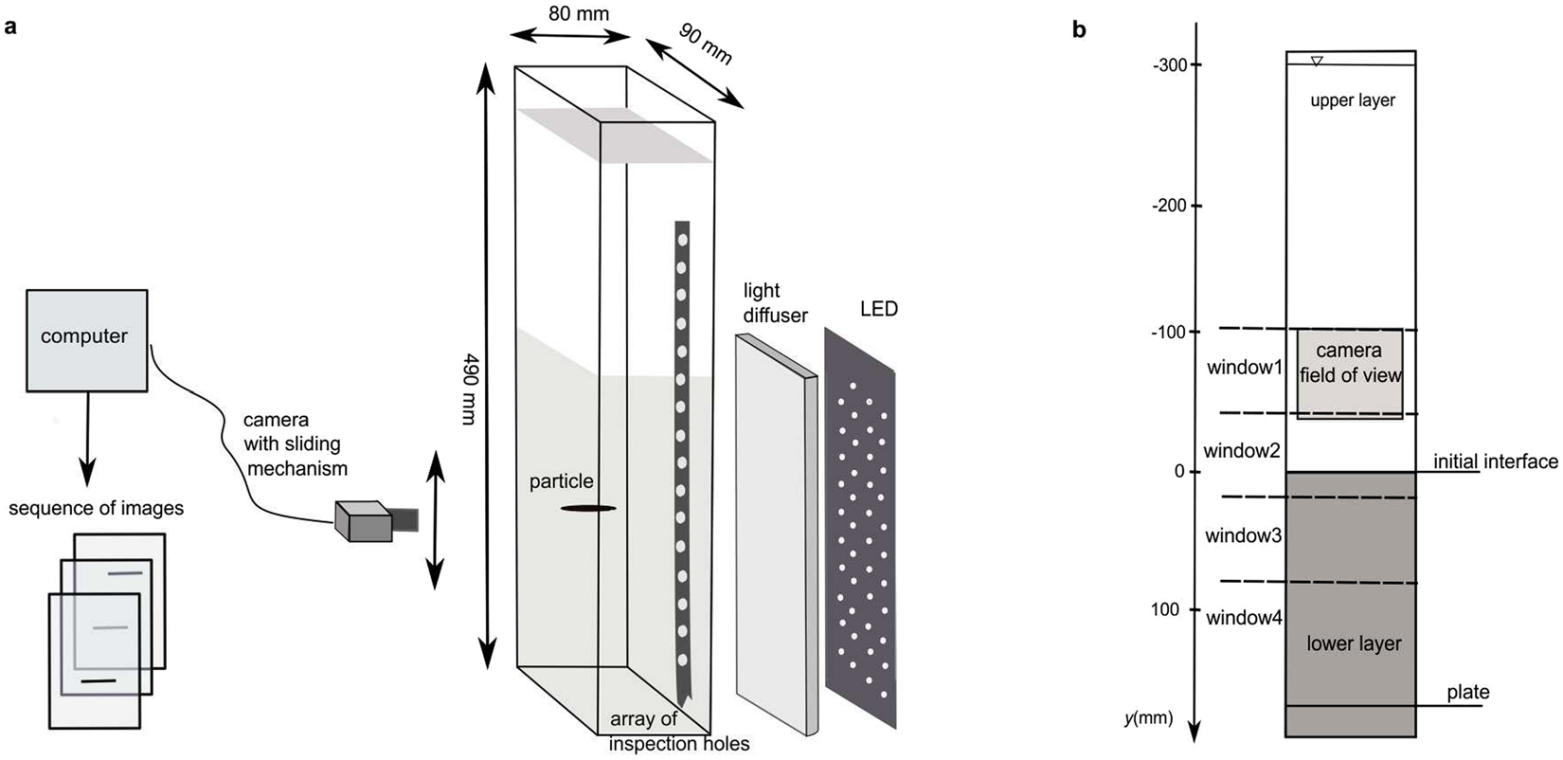
Schematic of experimental set-up. (**a**) Elements of set-up (not to scale). (**b**) Sketch of the front wall of settling tank. Vertical coordinates, location of reference windows, layers of liquid, and extent of camera field of view are shown. Detailed explanation provided in Methods.

Settling experiments were prepared and performed in the Laboratory of Hydrodynamic Micromodels at the Institute of Geophysics, Polish Academy of Sciences in Warsaw, Poland. Experiments were conducted in a specially designed settling tank (Fig. 1). A series of four experiments with density transition were performed by varying salinity, *S* of lower layer (1%, 3%, 5%, 6.4%), while upper layer was always freshwater. A density jump between upper and lower layer, *b* = (*ρ*_ll_ − *ρ*_ul_)/*ρ*_ul_, where *ρ*_ll_, *ρ*_ul_ are densities of lower and upper layer, ranged between 0.007 and 0.045. For each density jump, two experimental sub-sets were performed. They were different in the thickness of density transition, *L*_t_, and consequently in the strength of stratification defined by buoyancy frequency, *N*. Experimental sub-sets were named after experimental conditions characterised by the values of salinity of the lower layer, *S* and maximum buoyancy frequency, *N*_max_ − E*S*%*N*_max_.

The thickness of density transition varied from 28 mm to 83 mm, which is at least two orders of magnitude smaller than the thickness of pycnocline in natural waters. Since the ratio of the thickness of the density transition to particle diameter ranged from 15 to 46, the experimental density transitions may be considered a continuous stratification^22^. The density variation within transition was non-linear, and buoyancy frequency first increased with a depth from zero to maximum value *N*_max,_ which ranged over the interval (1.46, 6.13), and then decreased to zero again (Supplementary Fig. S2). Such strong stratification is not common in environmental fluids and may be referred only to sharply-stratified fjords and sinkholes; however, may be encountered in technical and engineering applications.

**Figure 2:**
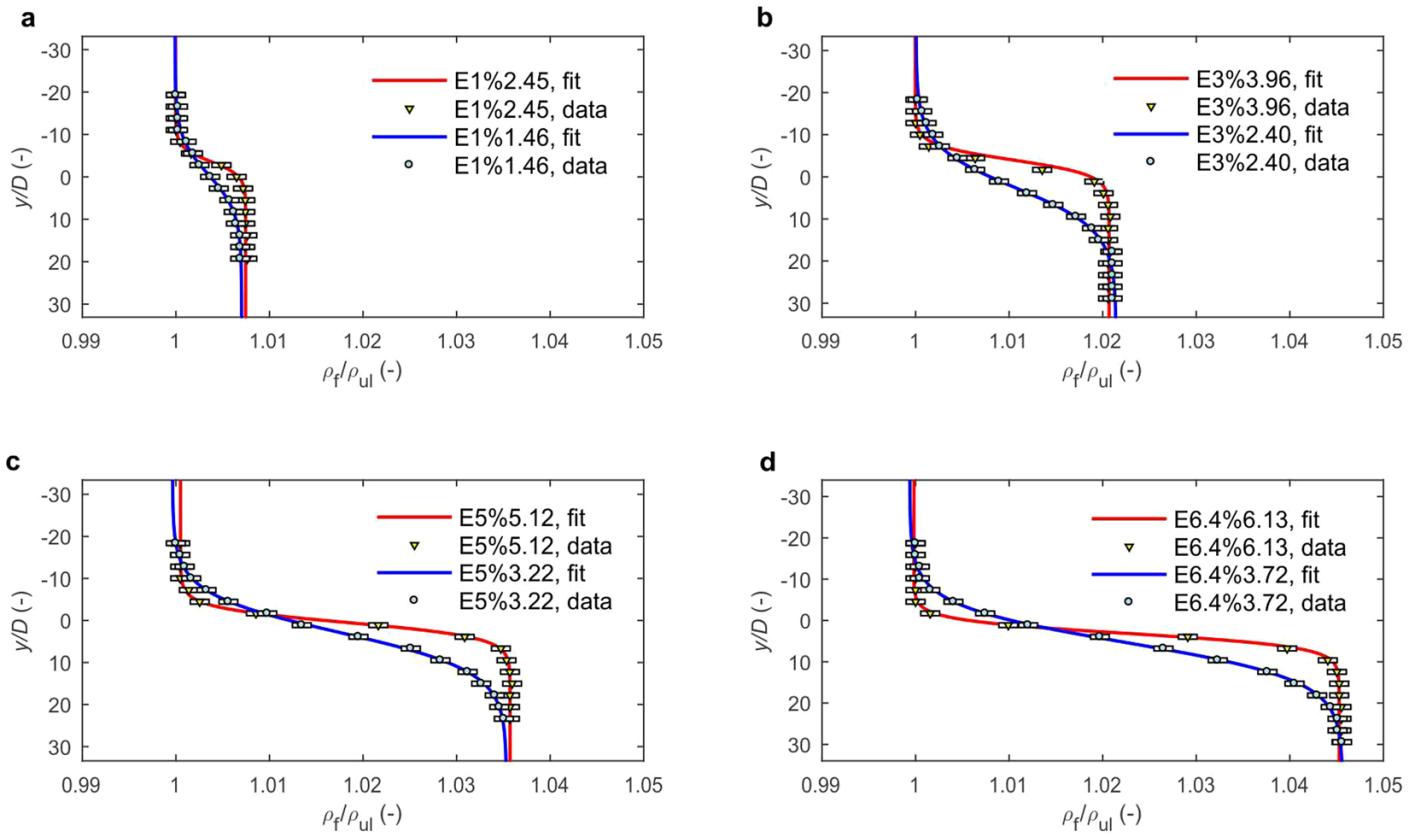
Density profiles in settling tank during experiments with ambient density transition. Figures show measured density profiles and the results of data fitting with Eq. (1). Measurement uncertainties are indicated as error boxes. Vertical coordinate is non-dimensionalised by mean disk diameter, *D* and liquid density by density in upper layer, *ρ*_ul_. *y/D* = 0 corresponds to theoretical position of density interface (see Fig. 1b).

Several settling tests were performed for each experimental sub-set using disks with diameters of about 1,800 μm and a density of 1,050 kg m^−3^ (Table 1). Density profiles during experiments are shown in Fig. 2. During each experimental test, the settling parameters of individual disks were measured. Before examining the settling, a particle was imaged by a digital microscope. Next, the particle was transported by a pincette and placed beneath the water surface in the settling tank. The particle floating down in the settling tank was visualised and recorded by a camera. Additionally, a settling experiment in homogeneous freshwater was carried out to evaluate the terminal velocity of particles in the upper layer, since in the experiments with density transition measurement data series in the upper layer were too short to evaluate the settling velocity. The detailed procedure is described in the Methods section.

**Table 1:** Description of settling experiments with density transition, physical conditions of liquid and characteristics of particles.

Experiment E*S*%*N*_max_	No. of tests/particles	*D*_ ± _SD (μm)	*T* (°C)	*ρ*_ll_ (kg m^−3^)	(*ρ*_p_ - *ρ*_ll_)/*ρ*_p_ (−)	*b* (−)	ν 10^−6^ (m^2^ s^−1^)	ν_ul_/ν_ll_ (−)	*L*_t_/*D* (−)	Fr_max_ (−)
E1%2.45	5	1791 ± 110	20.7	1,005.1	0.043	0.007	0.9029	0.97	15.6	0.60
E1%1.46	8	1827 ± 124	21.1	1,005.1			0.9029	0.97	40.8	0.98
E3%3.96	7	1806 ± 66	20.1	1,019.5	0.029	0.021	1.012	0.97	16.1	0.37
E3%2.40	3	1788 ± 112	20.2	1,019.4			1.011	0.97	46.4	0.61
E5%5.12	9	1805 ± 164	20.7	1,033.8	0.015	0.036	1.010	0.96	16.6	0.28
E5%3.22	8	1788 ± 175	20.5	1,033.9			1.014	0.96	42.8	0.46
E6.4%6.13	4	1768 ± 81	21.9	1,043.6	0.006	0.045	0.9114	0.79	15.0	0.24
E6.4%3.72	7	1764 ± 121	21.6	1,043.7			0.9960	0.94	41.7	0.40

*S* – salinity (%), *N*_max_ – maximum buoyancy frequency, *D* – mean particle diameter (see Supplementary Fig. S3), SD – standard deviation, *T* – temperature of water, *ρ*_ll_ – density of lower layer, *ρ*_p_ = 1,050 kg m^−3^ – density of disk, *b* – density jump evaluated as (*ρ*_ll_ − *ρ*_ul_)/*ρ*_ul_ where *ρ*_ul_ = 998.8 kg m^−3^ constant for all experiments, *ν*_ul_, *ν*_ll_ – viscosity of upper and lower layer, respectively, *ν* – reference viscosity evaluated as *ν* = (*ν*_ll_ + *ν*_ul_)/2, *L*_t_ – transition thickness defined as region with *N* > 0.2 (see Supplementary Fig. S2), Fr_max_ = *U*_ul_/*N*_max_*D* – maximal Froude number, where *U*_ul_ – terminal settling velocity in upper layer.

To make results more generic, they are non-dimensionalised. A diameter of disk, *d*, and thickness of transition layer, *L*_t_, are used as typical length scales. Velocity results are non-dimensionalised by terminal settling velocity in the upper layer or in the lower layer. It should be noted that several tests were performed for the same experimental conditions, and measurements of velocity, distance fallen in distinct settling phases and particle diameter were averaged over all tests in each experimental sub-set (stronger or weaker stratification conditions). To distinguish between the results of individual tests and averaged values, lower case letters (e.g., *u* for velocity, *d* for disk diameter) and capital letters (e.g., *U*, *D*) are used, respectively. Details on the statistical analysis of data is provided in Supplementary Materials.

### Pattern of disk evolution

**Figure 3:**
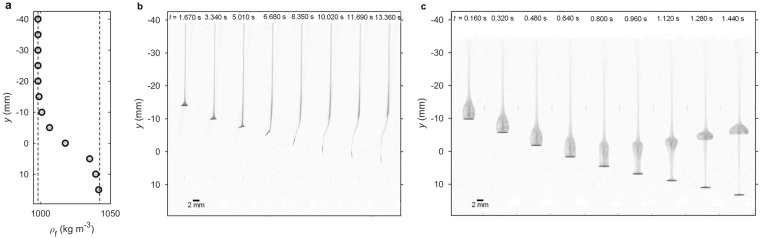
Evolution of disk entering ambient density transition at different Re number (qualitative illustration). (**a**) Vertical variation of ambient density. (**b**) Disk made of ABS (acrylonitrile-butadiene-styrene), *ρ*_p_ = 1,050 kg m^−3^; *d* = 2 mm, settling at Re ~ 10, (**c**) Disk made of PVC (polyvinyl chloride), *ρ*_p_ = 1,400 kg m^−3^, *d* = 3 mm, Re ~ 100. Images equally spaced in time. Particles were immersed in food colorant to enable observation of wakes behind them, here in grayscale. ABS particle starts to reorient to vertical position just after entering the density transition and entrains a tail of caudal fluid. PVC particle does not modify its orientation and large portion of wake of upper-layer fluid is separated just after crossing the transition due to buoyancy effect.

When non-spherical particles settle through a density transition, a reorientation of particle due to stratification may occur. It is possible only when inertial effects are low enough to let stratification-induced torques overcome pressure-induced torques. Figure 3 shows a comparison between the behaviour of disks settling through a density transition at two Re number regimes. Reorientation at density interface occurs only for a disk settling in upper layer at lower Re (Re ~ 10). In the experiments performed in this study, disks settle in upper layer with Re = 4.7 and during further settling Re does not exceed 5.5 (Supplementary Fig. S1). In such conditions reorientation occurs at density interface.

**Figure 4:**
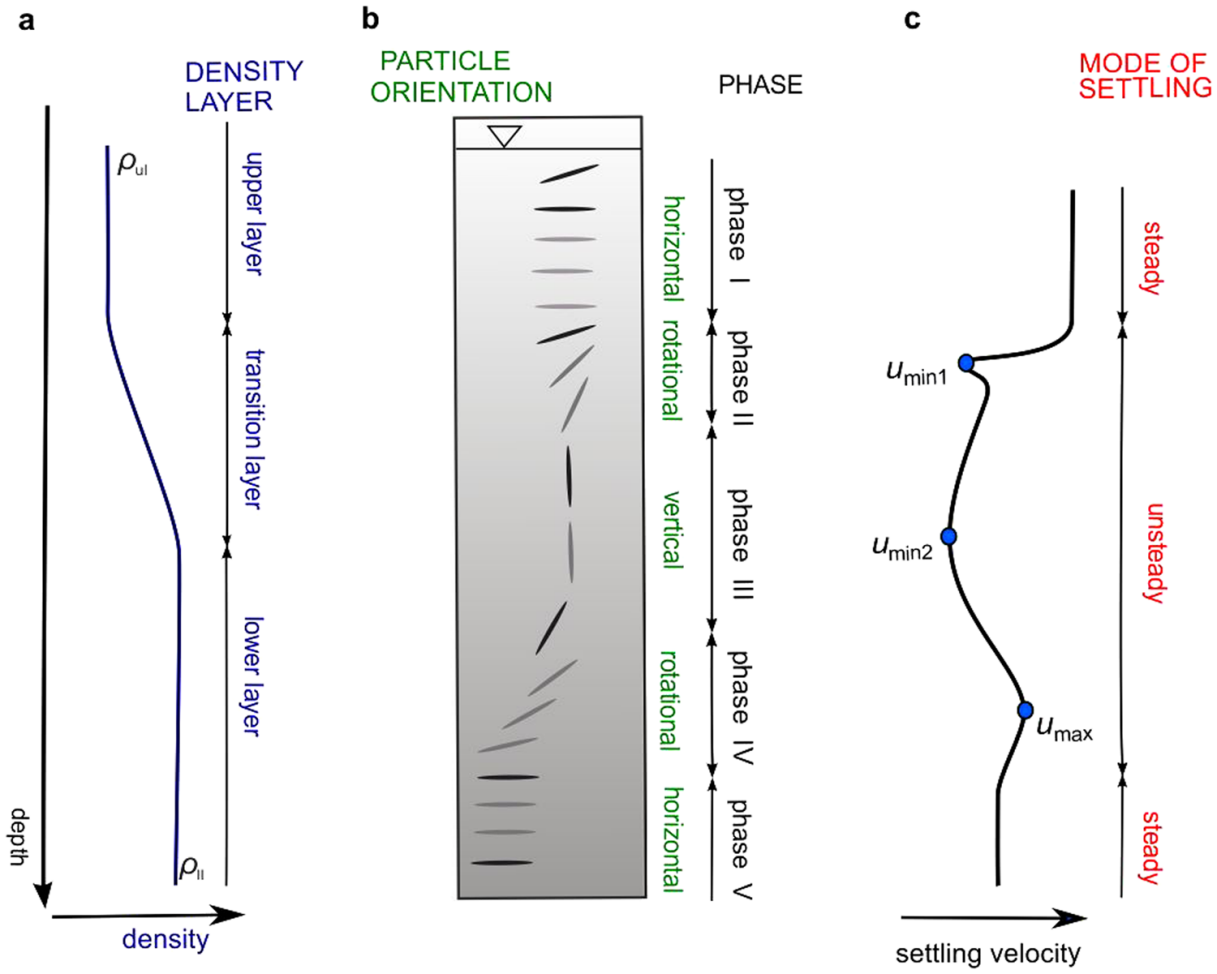
Schematic of disk settling through a column of two-layer liquid with density transition. (**a**) Vertical variation of density in the settling tank. Upper layer (ul) and lower layer (ll) contain liquid of homogeneous density, *ρ*_ul_ < *ρ*_ll_, density in a transition layer increases with depth. (**b**) Disk settling in the column of liquid with density variation defined in (**a**). Settling of disk is divided into five phases varying in orientation (angular position with respect to horizontal). (**c**) Variation of settling velocity. In phase I and phase V, the disk achieves terminal velocity when homogeneous layers are thick enough. In phase II, phase III, and phase IV the disk settle in unsteady motion with non-monotonic velocity variation. *u*_min1_, *u*_min2_, and *u*_max_ indicate characteristic velocities analysed in the text.

**Figure 5:**
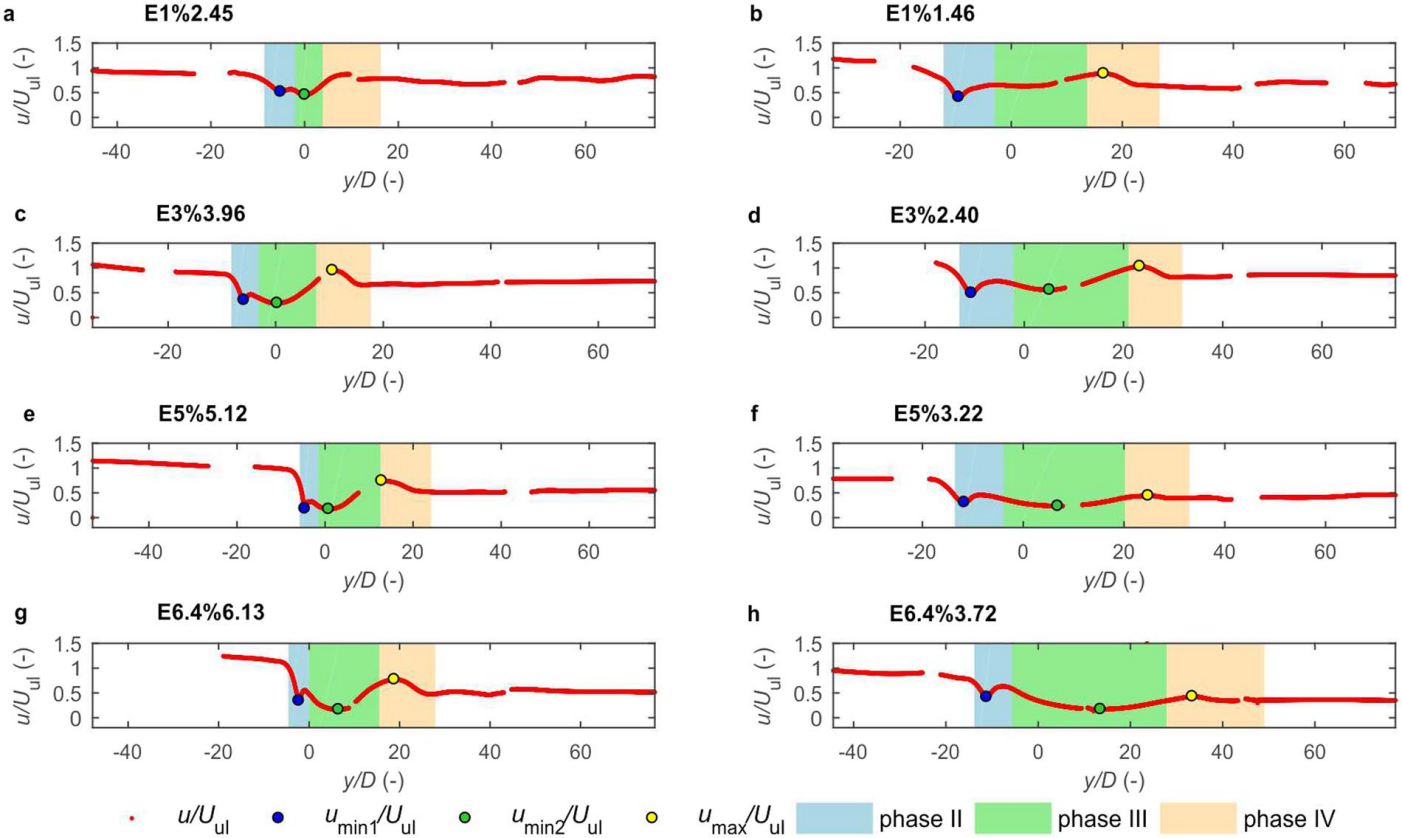
Variation of settling velocity for sample individual tests from each experiment. Variation of settling velocity is shown together with location of characteristic velocities *u*_min1_, *u*_min2_, *u*_max_ and vertical location of three unsteady settling phases – phase II, phase III, and phase IV. Settling velocity is non-dimensionalised by terminal settling velocity in upper layer, *U*_ul_, and particle position by mean disk diameter, *D*. *y/D* = 0 corresponds to the theoretical position of density interface (see Fig. 1b). Intermittency of velocity plots is due to the measurement method (explained in Methods).

I discovered a particular pattern of disk evolution when settling at a low Re number in two-layer liquid with continuous density transition. Figure 4 presents a diagram of particle behaviour and Fig. 5 shows sample results of settling velocity variations for individual experimental tests. As shown in Fig. 4b five settling phases may be specified. In phase I, the disk behaves as previously observed^44^, after being released in the homogeneous quiescent liquid, the disk immediately rotates to broadside aligned with the horizontal irrespective of initial inclination. This is caused by inertia associated with pressure-induced torques acting at points of stagnation. This falling style is one of a few falling regimes of disks identified so far^45^.

In phase II, a disk encounters the density transition, starts to rotate and decelerates to achieve the first local minimum velocity (*u*_min1_ in Figs 4c and 5). The disk rotates until it assumes a vertical position. It was demonstrated numerically for ellipsoid that reorientation in stratified ambient occurs due to buoyancy-induced torques, which counteract pressure-induced torques^21^. The particle decelerates just after entering transition due to the effects described in the introduction - added drag imposed by stratification and the buoyancy of caudal fluid entrained by a particle from the upper layer (entrained fluid is seen in Fig. 3b). Velocity measurements revealed local maximum velocity just after *u*_min1_ (Fig. 5), which is the effect of drag variation when particle rotates. As may be observed in Fig. 3b, the incidence angle (angle between the particle major axis and the direction of relative fluid velocity) varies from 90° and 0°. It was shown that drag decreases with the decrease of incidence angle for low Re numbers^36^. Based on that, the local acceleration of a disk may be explained by drag reduction due to the settling orientation of the particle.

In phase III, the disk settles in a vertical position and experiences deceleration and acceleration with the second local minimum velocity between (*u*_min2_ in Figs 4c and 5). Vertical position is retained by stratification-induced torques and the particle keeps settling in this position as long as ambient stratification is present. The non-monotonous velocity in the transition region is associated with non-linear stratification. In previous studies it was demonstrated that stratification-induced drag increases with stratification strength^22,23,28^. Particles tend to decelerate with increasing stratification strength assuming minimum velocity just after maximum stratification strength (Supplementary Fig. S2). In the region of decreasing *N*, the particles accelerate due to smaller drag and achieve velocity close to that of the lower layer.

When the effect of stratification fades, the pressure-induced torques take over, the disk rotates to broadside position and phase IV starts. The style of rotation is different from that in phase II and from the one observed at the beginning of phase I. Reorientation takes longer and is accompanied by a gliding motion, which may be associated with the fading effect of stratification and caudal fluid attached to the disk^18^. When particle settles in a low Re number regime, the mixing of caudal fluid with ambient water is slow, and lighter fluid attached in the upper part of the particle may oppose inertial effects. The disk accelerates during gliding motion achieving local maximum velocity *u*_max_ (Figs 4c and 5). This acceleration is consistent with the settling behaviour of disks falling in gliding motion reported for higher Re numbers^35^. The acceleration of particle is explained by the decrease of incidence angle. In the lower layer, the particle assumes stable horizontal position, which is classified as phase V.

The study shows that a disk settling in considered stratification conditions exhibits a more complicated pattern of settling velocity variation than the studied experimentally to date, solid spheres^18–20^ porous spheres^27,46^ and laboratory formed marine snow aggregates^31^ settling in two-layer fluids. While the aforementioned particles achieve one minimum velocity after crossing a density interface, a disk may achieve two local minimum velocities in the density transition region – *u*_min1_ and *u*_min2_. Moreover, a disk achieves two local maxima. The occurrence of the first minimum velocity just after entering density transition is common for this experiment and the previous ones. However, the second velocity minimum and two local maxima are specific for this study. While the acceleration of particle during reorientation is affected by the incidence angle and is specific to the shape of the particle, the second minimum in transition layer is affected by non-linear stratification and associated drag variation. Such stratification-induced local minimum should also occur for spherical particle settling in similar stratification conditions, which will be verified in another study.

### Impact of density transition on disk settling behaviour

**Figure 6:**
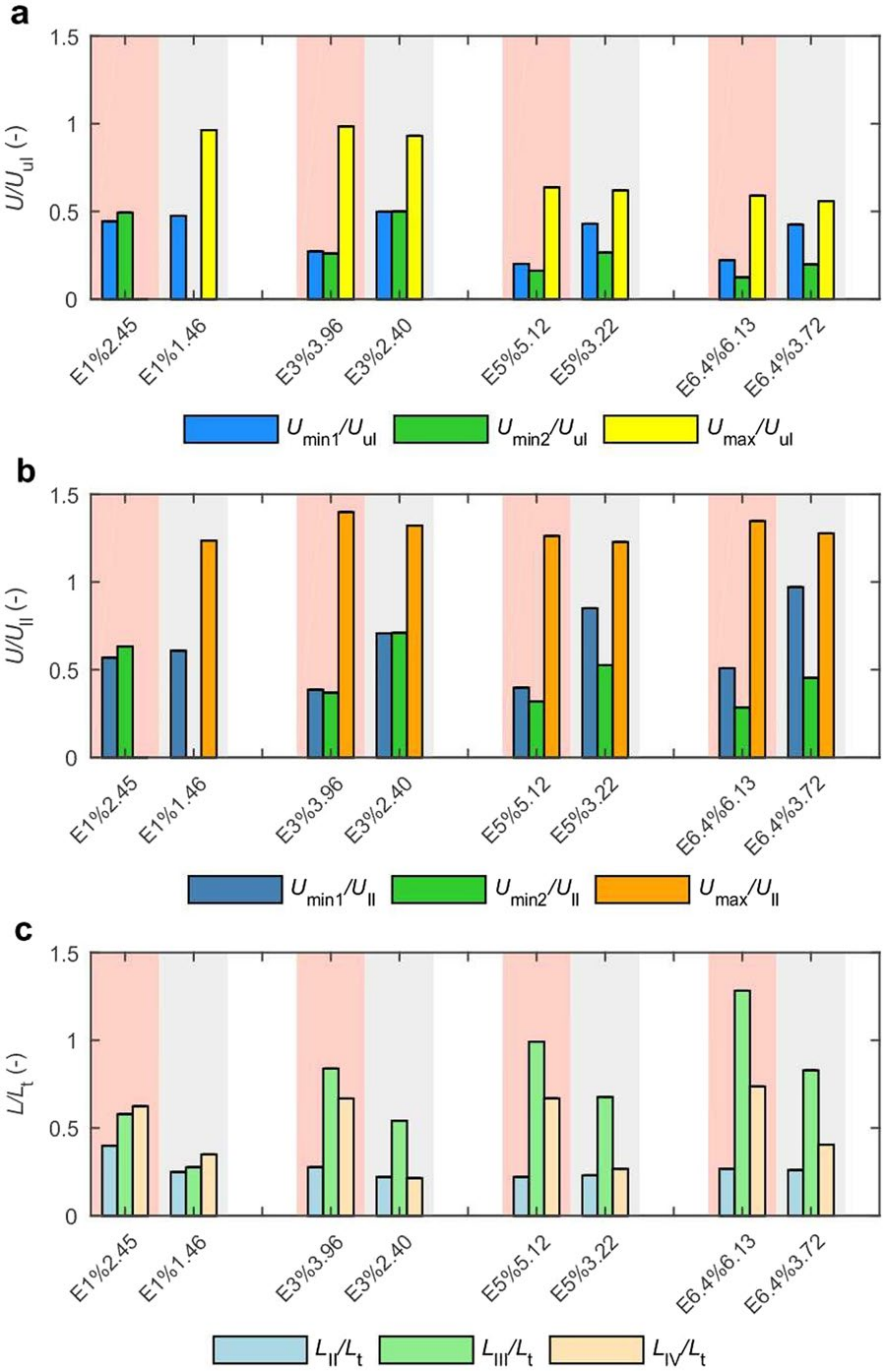
Comparison of disks settling dynamics for different conditions of density transition. (**a**,**b**) Bar graphs represent mean characteristic velocities *U*_min1_, *U*_min2_, and *U*_max_ (averaged over experimental tests) non-dimensionalised by terminal settling velocities in upper (*U*_ul_) and lower layer (*U*_ll_). Supplementary Fig. S5 provides data for evaluation of mean values. Details on *U*_ul_ and *U*_ll_ evaluation are given in Methods and Supplementary Fig. S4. (**c**) Bar graphs represent mean vertical distance fallen in three phases (*L*_II_, *L*_III_, *L*_IV_) non-dimensionalised by thickness of transition layer *L*_t_. Supplementary Fig. S6 provides data for evaluation of mean values.

This experimental study shows that the stratification strength and the buoyancy jump are parameters that affect the behaviour of disks settling in two-layer ambient with density transition. The effect of increasing the buoyancy frequency of the transition layer on the dynamics of particle settling is assessed by comparing the results of experiments with the same density jump and varying stratification strength. The results presented in Fig. 6a,b show that the stratification strength of the transition layer affects both local minimum velocities. The velocities are suppressed to a larger extent compared to the velocity in the upper layer in sub-sets with stronger stratification (higher *N*_max_) than in sub-sets with a weaker stratified transition. This may be explained by the effect of added drag due to stratification. As was previously shown, the drag increases with stratification strength^19,23,25^, which is evident for experiments E3%, E5% E6.4%. The first minimum velocity is as much as four times smaller than *U*_ul_ for sub-sets with stronger stratification and about two times smaller than *U*_ul_ for weaker stratification. The difference between stratification strength for the experimental sub-sets with the smallest density jump (E1%) was too small to result in a significant difference in the first minimum velocity; *U*_min1_ is two times smaller than the settling velocity in the upper layer for both experimental sub-sets.

In previous experiments with spheres and aggregates, it was observed that the minimum velocity was usually smaller than the velocity in a lower layer^18–20,27,31,46^. Although in this study the transition layer is thicker than in mentioned studies, the results show that drag due to stratification is strong enough to decrease particle velocity below settling velocity in the lower layer. As demonstrated in Fig. 6b, both minimum velocities are lower than *U*_ll_, with *U*_min1_/*U*_ll_ ranging between 0.39 and 0.97 and *U*_min2_/*U*_ll_ between 0.28 and 0.71. Since *U*_min1_ is not affected by the density of the lower layer to the same extent as *U*_min2_, for weak stratification conditions and a higher density jump between the layers (as in E6.4% 3.72) it is likely that *U*_min1_ could exceed *U*_ll_.

Stratification strength is a combined effect of the magnitude of density variations between the upper and lower layer and transition thickness. Generally, the larger density jump, the stronger stratification is possible, which is additionally inversely proportional to the thickness of the transition layer. In this study, stratification strength was decreased by extending the thickness of transition while keeping the same density conditions in the upper and lower layer. However, stratification strength may be also controlled by varying the density jump and keeping the thickness of transition constant. In both cases, the magnitude of density variations between the upper and lower layer affects the stratification strength that may be achieved, and thereby settling behaviour within the stratified transition layer. While the effect of the density jump on settling dynamics is evident for thin density transitions^18–20^, the effect of stratification seems to be more significant than the density jump in explaining the settling behaviour of particle when the thickness of transition much exceeds the dimensions of the particle, which is the case in this study.

In the experiments, the density variation between homogeneous layers was achieved by changing only the density of the lower layer. The impact of lower layer density on settling dynamics is seen in the maximum velocity occurring in phase IV. No matter how much particle decelerated in the transition layer and how small the second minimum velocity was, reorientation during phase IV was accompanied by significant acceleration, and disks locally achieved velocities higher than the settling velocity in the lower layer. Averaged data presented in Fig. 6b demonstrate that *U*_max_ is about 1.2–1.4 higher than the terminal velocity in the lower layer. Since the maximal velocity is achieved in the lower layer or in the lower part of transition (Supplementary Fig. S2), the density of the lower layer markedly affects the maximal velocity. When *U*_max_/*U*_ul_ in Fig. 6a are compared between experiments with different density jumps (which in this study is equivalent to different densities of the lower layer) it is seen that the maximum velocity decreases with increasing the density of the lower layer.

The impact of the density variation between layers on settling behaviour is also seen in the distance fallen in distinct settling phases. Figure 6c shows that distance fallen in a vertical position relative to the thickness of the density transition, *L*_III_/*L*_t_ increases with the density jump, while the distance of the first and second reorientation are not as sensitive to the density variation between the layers. When subsets with the same density jump are compared, it is seen that *L*_III_/*L*_t_ and *L*_IV_/*L*_t_ are smaller for weaker stratification conditions. This may be explained by the impact of the lighter fluid from the upper layers attached to the particle, which modifies pressure distribution around a particle. The role of caudal fluid may be supported by the fact that *L*_III_/*L*_t_ increases with the density of the lower layer, which means that a particle falls a longer distance in a vertical position when the density of particle is closer to the density of the lower layer. The particle settles with lower velocity and the boundary layer of lighter fluid may persist longer due to less intensive mixing of the attached fluid with the ambient^18^. Additionally, the particle experiences larger deceleration for stronger stratification conditions, and the mixing of attached fluid with the ambient is slower than in weaker stratification, and lighter fluid may oppose rotation in a longer distance relative to transition thickness.

The settling behaviour presented in this study is specific to the continuous stratification of the transition layer, that is the transition thickness larger than the particle dimensions. It may be expected that some of the described settling behaviours may not occur or may be modified for thinner transitions. If the thickness of transition was on the order of particle diameter, the effect of non-linear stratification strength may not be significant and consequently the stratification-dependent second minimum velocity may not occur. In such case, only the first minimum would be achieved as in other studies with thin transitions^20,27,31,40^. Reorientation of disk may also be modified. I suspect that disk would rotate but will not achieve vertical position before exiting the thin transition, as was in the case of the ellipsoid described in another study^21^. Starting from such an inclined position, the particle would glide in the lower layer to achieve a stable horizontal orientation. These comments are based on observations made for salt as a stratifying agent, however, it should be noted that the settling behaviour would be different for other stratifying agents, e.g., temperature, due to agent diffusivity.

Disks are only one kind of non-spherical particles that may undergo stratification-induced reorientation affecting their settling dynamics. I hope this paper will encourage extending the experimental and numerical studies to other shapes of non-spherical particles and more complex stratification conditions. Further experimental and numerical studies are necessary to extend the dataset that will allow to answer further detailed questions that arise from this research. This study was focused on evaluating the effects of characteristics of density transition on the settling behavior of disk, and only the transition characteristics were varied, while parameters such as the density of the upper layer, and the density and dimensions of the particle, were kept constant. Further studies may consider variable parameters to analyze how the Re number in the upper layer affects the settling behavior in density transition. Moreover, experimental conditions may be extended to consider a wider range of stratifications, transition thickness, and density jumps.

## Methods

### Settling tank with density stratification

The settling experiments were conducted in a specially designed rectangular tank measuring 0.490 m in height with transparent polycarbonate side walls (Fig. 1). The interior dimensions of the tank were 0.090 m × 0.080 m. When compared with the dimensions of the particles, the tank was wide enough to eliminate wall effects. Salt (NaCl) water solution was prepared using distilled water. The water had the same room temperature as the facility to prevent any convection currents due to temperature gradients during the experiments.

A two-layer water column was formed in the tank, comprising salty lower layer and freshwater upper layer. The initial density interface was located at 0.190 m from the bottom. Vertical coordinates were set so that zero corresponded to the position of the theoretical interface (Fig. 1b). To form the interface, a plate with small holes was installed on a horizontal plane at 0.020 m from the tank’s bottom. Salt water solution was poured beneath the plate up to its level. Next, freshwater was poured from upstream using a diffuser constructed from a sponge. The plate prevented the mixing of the salt water solution with freshwater. When the tank was filled with the desired volume of freshwater, filling with salt water solution was restarted and lasted until the water column reached a height of 0.480 m. The filling took about two hours, and the filling rate was controlled to prevent air bubbles forming. A transition layer with density gradient formed between the lower and upper layer due to mixing during the filling procedure. To determine the vertical variation of salinity and the density in the tank, samples of liquid were taken using a vertical array of inspection holes spaced at a distance of 5 mm. About 1 ml of liquid was withdrawn from an inspection hole by using a medical syringe with a 20G(0.9 × 40) needle. Holes were secured with silicone rubber which enabled smooth injection and prevented from leaking after the needle was removed from the opening. Salinity of each sample was measured to an accuracy of 0.1% using a digital Kruss refractometer, model DR301-95. The water temperature was controlled to an accuracy of 0.1 °C using a liquid thermometer. During each day of experiment, the vertical variation of salinity and temperature were recorded every hour, three times in total, then the results were averaged. Variation in temperature during one experimental day did not exceed 0.6 °C. Given the information about salinity and temperature, the density and viscosity was evaluated based on the tables published in the literature^47^. Values of viscosity are given in Table 1.

The variation of liquid density with depth was fitted to the hyperbolic tangent function^31^:

1 $${\rho }_{f}(y)=(\frac{{\rho }_{ll}-{\rho }_{ul}}{2})(1+\,\tanh (\frac{y-{y}_{0}}{z}))+{\rho }_{ul}$$

where *ρ*_f_ - density of ambient liquid (kg m^−3^), *ρ*_ul_ and *ρ*_ll_ - density of upper and lower homogeneous layer, respectively (kg m^−3^), *y* – vertical coordinate (m), *y*_0_, *z* – fitting parameters (m). Results of measured and fitted density profiles for all experiments are presented in Fig. 2. Density profiles were fitted to the tangent function with R^2^ > 0.99.

Bundt-Vaisala buoyancy frequency, *N*, which is a measure of density stratification, was evaluated as a function of vertical coordinate by applying the following relation:

2 $$N(y)=\sqrt{\frac{g}{{\rho }_{f}(y)}\frac{\partial {\rho }_{f}(y)}{\partial y}\,}$$

where *g* = 9.81 m s^−2^ – acceleration due to gravity, $\frac{\partial {\rho }_{f}(y)}{\partial y}$- background density gradient at vertical coordinate *y*. Next, the results were fitted to the Gaussian function with R^2^ > 0.99. The results are shown in Supplementary Fig. S2.

### Preparation of particles

Particles were made of acrylonitrile butadiene styrene (ABS) with a density of 1,050 kg m^−3^. To prepare the particles, an ABS–acetone solution was prepared and a drop formed from the solution was released onto a polished glass plate. After the acetone evaporated, a thin disk was formed. Thickness was about 12 μm–15 μm and diameter to thickness ratio was greater than 120. Twenty particles were selected for experiments and in each experiment the same set of particles was used. However, the final number of particles analysed in each experiment was smaller (Table 1), since some tests had to be disregarded at different stages of the experimental process and data analysis. Particles are durable enough to be used several times in the experimental study.

Before a settling test, each particle was investigated by microscopic analysis. The particle was placed on a backlight table and was imaged by the COMOS digital microscope at a resolution of 640 × 480 pixels, where one pixel corresponded to 6.4 μm. A sample photograph is shown in Supplementary Fig. S3. To calibrate the microscope, several photos of the micrometre reading up to 0.01 mm were taken. Image analysis was performed using image processing tools available in MATLAB. Particle metrics, such as area, *A*, diameter – defined as circular equivalent diameter, *d*, perimeter, *P*, were evaluated. In experiments with density transition, the mean particle diameter, *D*, varied between 1,764 μm and 1,827 μm and circularity of particle (4 π *A*/*P*^2^) was greater than 0.99 indicating an almost circular shape (Supplementary Fig. S3). In experiments with homogeneous density, the mean particle diameter varied between 1,764 μm and 1,802 μm (Supplementary Fig. S4).

### Visualization of settling particle

To facilitate the visualization of particles settling in the tank, a shadowgraph (backlight method) was used^48^. A LED surface lamp was placed beneath the tank and a diffuser was placed between the lamp and the tank to ensure homogeneous illumination. The low energy LEDs prevented the lamp from heating the experimental facility and causing unwanted convective movement of the liquid. At the same time, a LED lamp is strong enough to effectively illuminate a particle.

The settling of a particle was recorded at an acquisition rate of 30 frames per second using the Basler acA2500-60um USB 3.0 camera with Schneider-Kreuznach macro lenses Componon 2.8/28–001 with aperture 3.5 F. 6 mm extension tube, which resulted in the field of view 77 mm × 62 mm, one pixel corresponded to 31 μm (see Fig. 1b). The camera was calibrated by using micrometre reading up to 0.01 mm. For the experimental conditions, the settling velocity of the particles was smaller than 4 mm s^−1^, which allowed for the required accuracy in the orientation and velocity evaluation, given the size of the particles, the acquisition rate, and the image resolution.

Since particle tracking distance was longer than the vertical dimension of the field of view, images of settling particle were taken in four windows marked by fixed horizontal lines spaced every 60 mm at the front wall of the settling column (Fig. 1b). A stable tripod, which enabled precise manual vertical movement of the camera in the range of 0.3 m, was used. The camera remained in a stable position until the particle was outside the window. Next, the camera was lowered to take the image in the next window. In case of a particle moving sideways, the tripod was equipped with a pivot table allowing for horizontal movement parallel to the front wall within a distance of 40 mm. Transverse movements of a particle were not analysed in this study.

The edge of the camera lens was positioned at a distance of 0.148 m from the front wall. This corresponded to the focal plane in the centre of the channel width. When a particle fell outside this plane, the camera could be moved back or forward in the range of (−30 mm, 30 mm) relative to the initial position. The operator focused the camera on a particle in the first window and the distance of the camera from the front wall was not changed afterwards. The information about the location of fixed lines on the front of the tank, and the camera’s backward or forward shift were used to map between the pixel vertical coordinate in an image and the real coordinate in the tank. For this purpose, calibration was performed before experiments. A ruler was placed inside the tank at different distances from the front wall. The ruler was adjusted to measure the distance from the column bottom. The camera was shifted backwards or forwards, accordingly, for each ruler position. Information about the camera shift combined with the information about real coordinates obtained for each window allowed to work out the mapping relationship. Uncertainty of the measured position was about 1 mm.

### Image analysis

At the pre-processing stage, background noise was eliminated by “rolling ball” background subtraction available in ImageJ with the radius equal to 200. Then digital filtering by the threshold method available with MATLAB tools was used to convert the images to binary mode. Since the experimental illumination conditions were stable during all experiments, the threshold was constant for all tests −0.9. During image analysis, it was sufficient to control the performance of these parameters only for a sample of the images.

At the processing stage, measurements were taken based on the geometrical properties of the 2D projection of the particle in the binary image by using MATLAB image-processing tools. The processing of images from settling tests comprised the morphometry analysis of particle projection necessary to evaluate the orientation of the particle and identification of the centroid of projection necessary to evaluate the settling velocity. The particle was identified in each image and tracked in a sequence of images originating from the same window. One path was identified in one window, and for the majority of tests four continuous data sets were obtained corresponding to each window (see Fig. 5). If there were some problems with particle performance in some images (e.g., poor contrast) then the particle projection was ignored and the path was divided into several pieces. The time step between consecutive images was derived based on the frame rate of the recorded video.

### Measuring velocity

Once the particle paths in four windows and the time step between the consecutive images were determined, the vertical component of particle velocity, i.e., the settling velocity *u* was evaluated for each experimental test. First, the Savitsky-Golay filter was used to fit a cubic polynomial to time-resolved particle position data. The Savitsky-Golay filter is available in MATLAB, and it applies a moving-average polynomial. Next, *u* was evaluated from filtered data as a central difference quotient, and the results shown in Fig. 5 were obtained.

The settling velocity in the homogeneous upper and lower layer was evaluated for data analysis. To measure the settling velocity in the lower layer, results of experiments with density transition were used (E1%, E3%, E5%, E6.4%). Terminal settling velocity was evaluated based on velocity measurements in window no. 4. For each experiment, *m* experimental tests with long enough series of relatively constant *u* in lower layer were chosen. For each chosen test, *u* was averaged over at least 350 data points to obtain *u*_lli_ where *i* = 1, ..., *m*. Next, terminal velocity in lower layer, *U*_ll_ was evaluated as ${U}_{ll}=\frac{1}{m}{\sum }_{i=1}^{m}{u}_{lli}$.

Since in the experiments with density transition measurement data series in the upper layer were not long enough to evaluate average settling velocity, *U*_ul_, a separate experiment on settling of disks in homogeneous freshwater was performed. Measurements were performed in the same settling tank and velocities were evaluated by using methods described above. Measurements from window no. 4 were used and the procedure for evaluating *U*_ul_ was the same as described above for *U*_ll_. Table 2 presents the details of settling conditions in homogeneous liquid and Supplementary Fig. S4 presents data used to evaluate average velocity comprising sample size, dimensions of particles and settling velocities.

**Table 2:** Physical conditions of liquid and characteristics of disks used to measure terminal settling velocity in homogeneous liquid. *S* – salinity, *D* – mean particle diameter, SD – standard deviation, *U* – mean settling velocity (see Supplementary Fig. S4).

S (%)	No. of tests/ particles	*D* ± SD (μm)	*U* ± SD (mm s^−1^)	Re (−)
0.0	14	1764 ± 121	2.62 ± 0.27	4.7
1.0	12	1802 ± 113	2.04 ± 0.14	4.0
3.0	10	1801 ± 76	1.84 ± 0.24	3.2
5.0	16	1799 ± 170	1.32 ± 0.22	2.3
6.4	12	1781 ± 113	1.14 ± 0.14	2.0

## Electronic supplementary material

Supplementary figures
