# Supplementary material for: Stratification-induced reorientation of disk settling through ambient density transition
Source: Sci Rep. 2018 Jan 11;8:412. doi: 10.1038/s41598-017-18654-7 (PMC5764956; doi:10.1038/s41598-017-18654-7)
Supplement: Supplementary file 1 — Supplementary figures [file 41598_2017_18654_MOESM1_ESM.pdf]

Supplementary Figures

Stratification-induced reorientation of disk settling through ambient density transition

Magdalena M. Mrokowska[1*]

[1*] Institute of Geophysics Polish Academy of Sciences, Ks. Janusza 64, 01-452 Warsaw, Poland

e-mail: m.mrokowska@igf.edu.pl

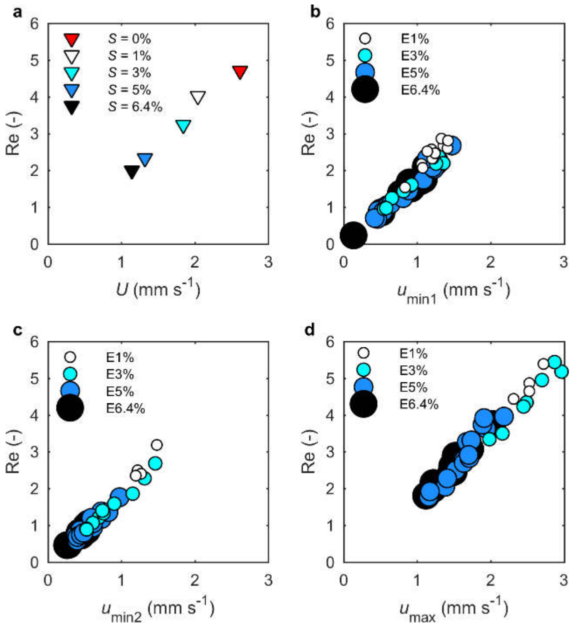

Supplementary Figure S1. Variation of Reynolds number with settling velocity. a, Settling with terminal velocity in homogeneous density conditions characterized by salinity, *S*. For details refer to Methods. **b-d**, Re number for instantaneous characteristic velocities in experiments with density transition.

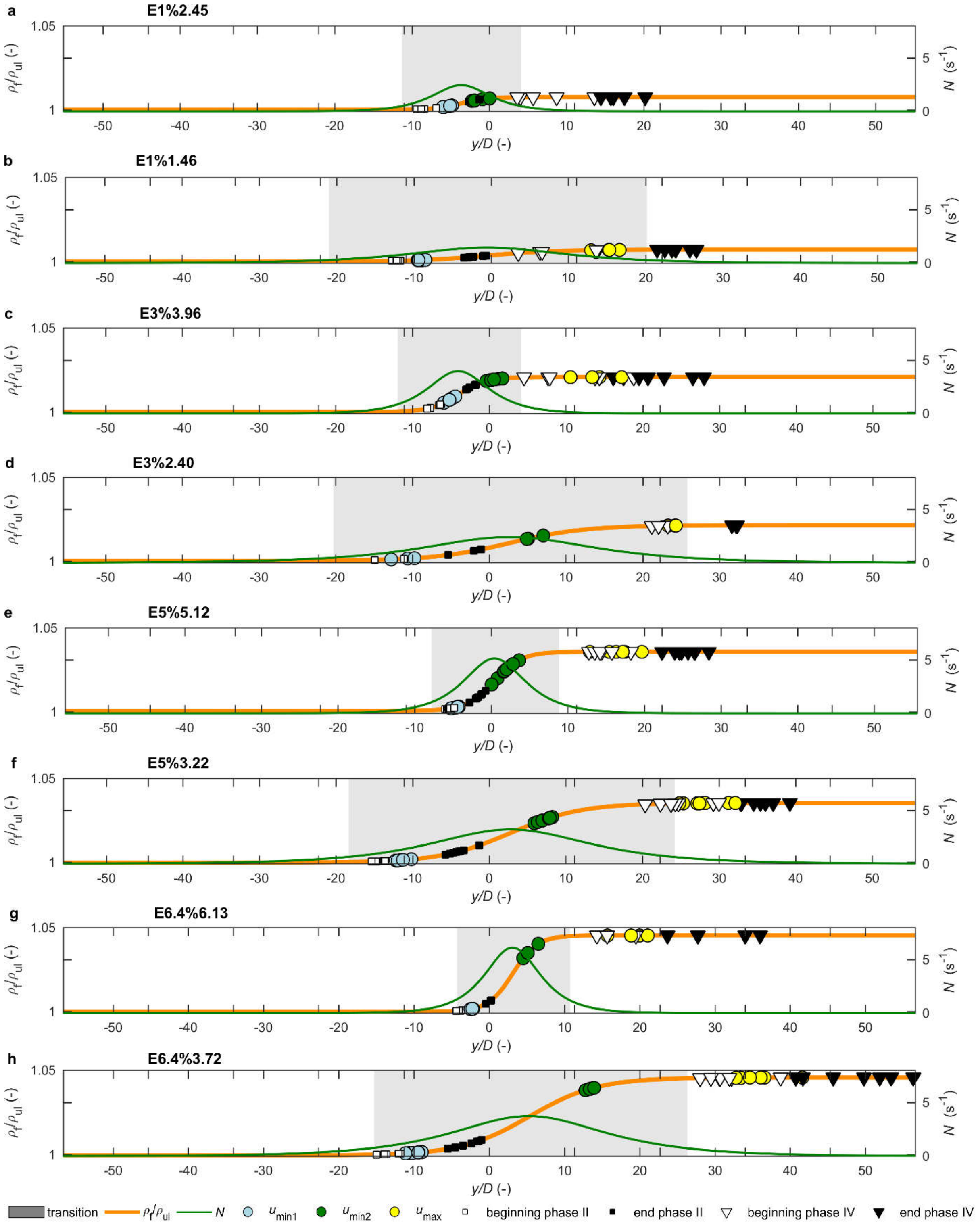

Supplementary Figure S2. Characteristic points and phases of disk evolution and stratification conditions in experiments with density transition. Stratification conditions are represented by the ratio of ambient density, ρ_f to the density in upper layer, ρ_{ul} and frequency buoyancy, N, as a function of non-dimensional position of particle where y – vertical coordinate and D – mead disk diameter. In grey – region occupied by density transition evaluated as $\rho_{ul} + 0.01(\rho_{ll} - \rho_{ul}) < \rho_f < \rho_{ll} - 0.01(\rho_{ll} - \rho_{ul})$.

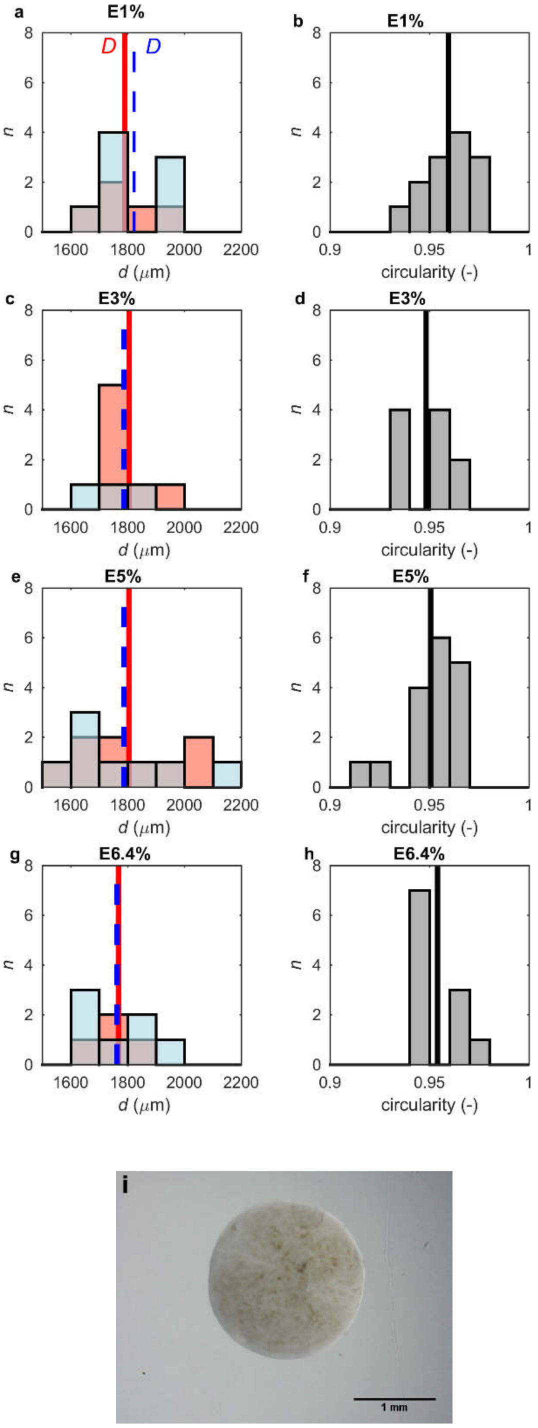

Supplementary Figure S3. Characteristics of disks used in experiments with ambient density transition. a-h, Histograms of diameter and circularity of disks. In red – results for sub-sets with stronger stratification, in blue – results for sub-sets with weaker stratification, in grey – results for all particles used in an experiment. **i**, microscope photograph of sample disk used in experiments. *n* – sample size, number of experimental tests, *d* – diameter of individual disk, *D* – diameter averaged over all tests in experimental sub-set. Standard deviations are given in Table 1.

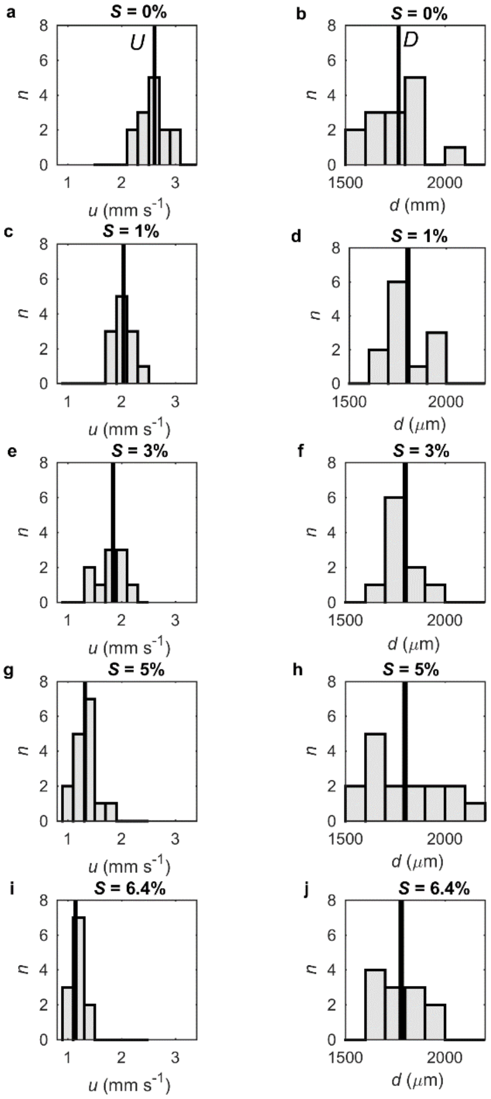

Supplementary Figure S4. Histogram of terminal settling velocities and diameters in tests in homogeneous density ambient liquid. **a-b**, Data for settling in homogeneous liquid of salinity S = 0%. **c-j**, Data for settling in salty water – results from experiments E1%-E6.4% for terminal settling in lower layer. n – sample size, number of experimental tests, u – settling velocities in individual tests, U – settling velocity averaged over all experimental tests, d – diameter of individual disk, D – diameter averaged over all experimental tests. Standard deviations are given in Table 2.

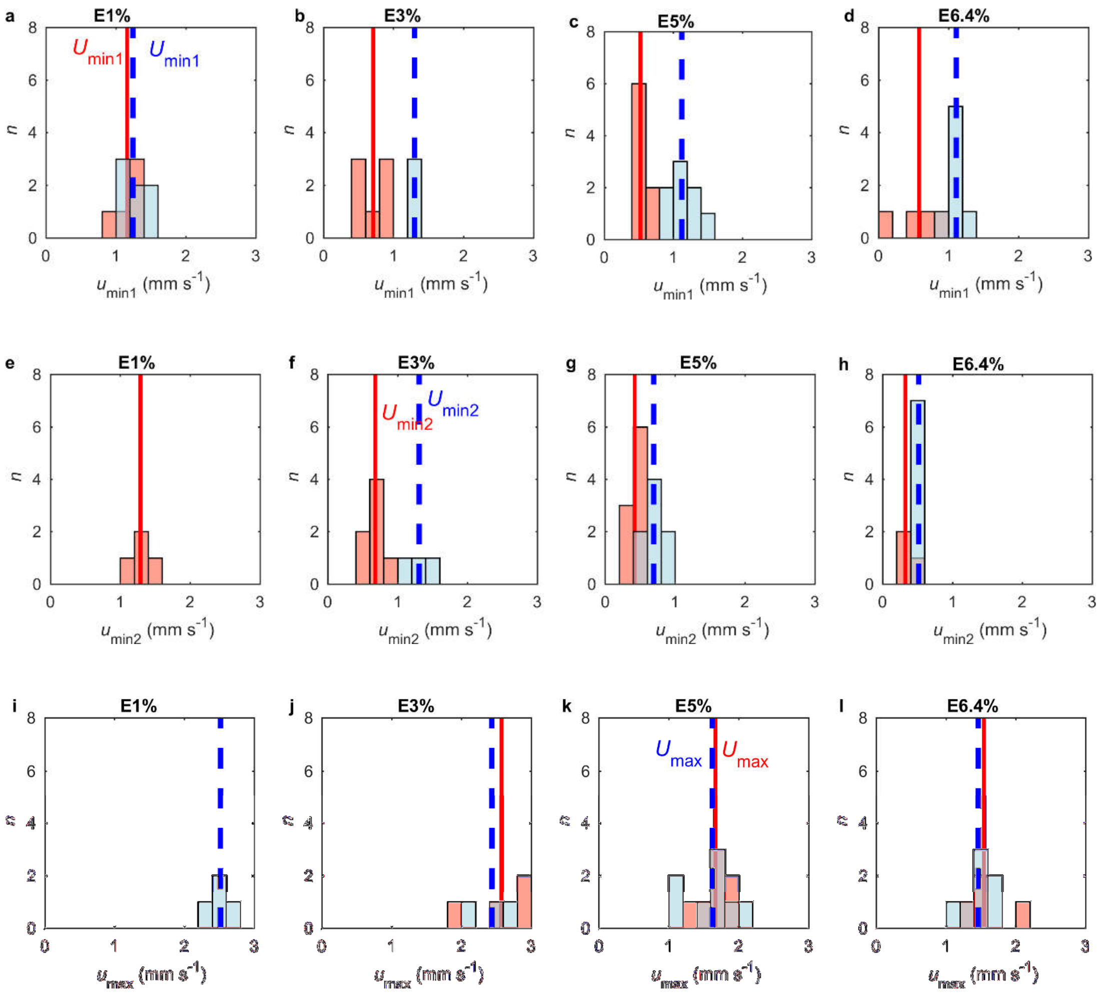

Supplementary Figure S5. Histograms of characteristic velocities in experiments with density transition. a-d, the first minimum velocity **e-h**, the second minimum velocity **i-l**, local maximum velocity. In red – results for sub-sets with stronger stratification, in blue – results for sub-sets with weaker stratification. *n* – sample size, number of experimental tests, *u* – settling velocities in individual tests, *U* – settling velocity averaged over all tests in experimental sub-set.

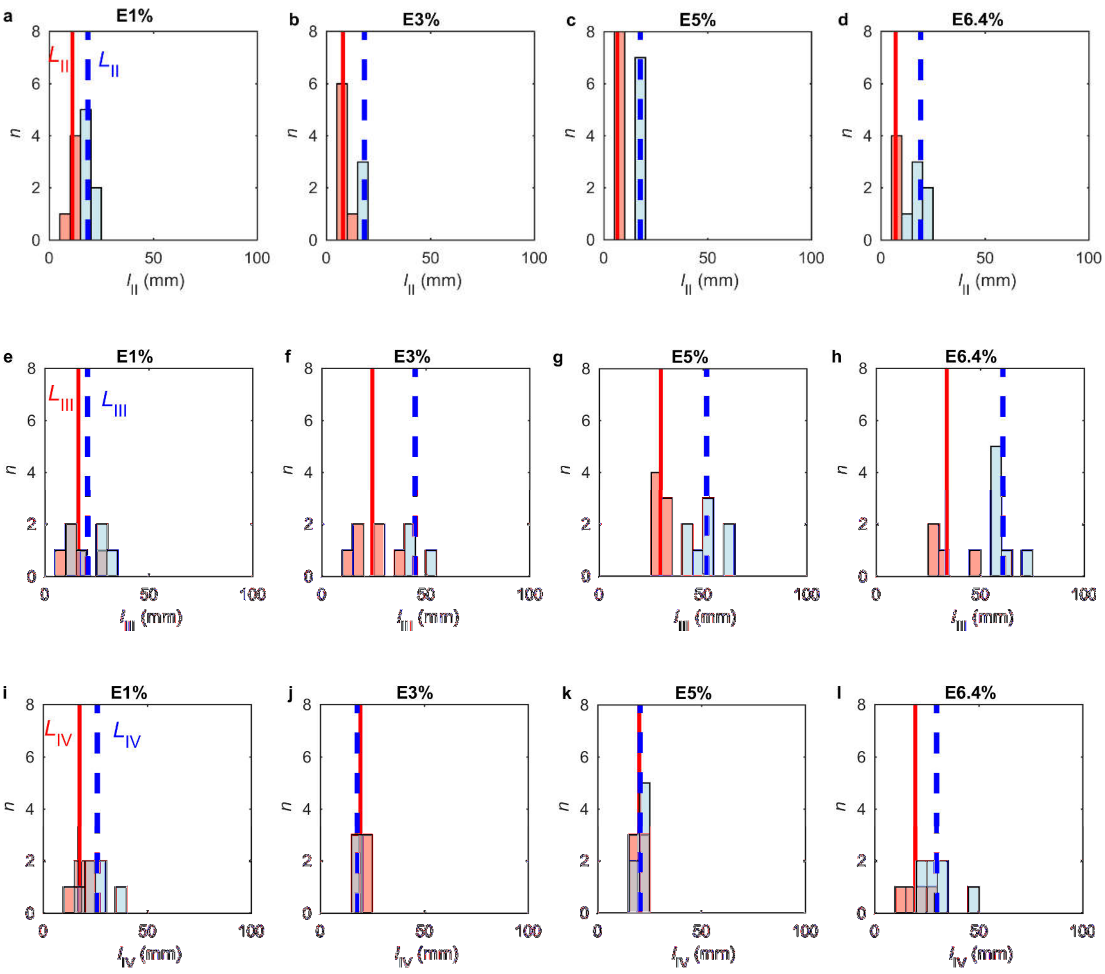

Supplementary Figure S6. Histograms of settling distance in distinct phases. a-d, phase II, **e-h**, phase III **i-l**, phase IV, In red – results for sub-sets with stronger stratification of density transition, in blue – results for sub-sets with weaker stratification of density transition. n – sample size, number of experimental tests, l – falling distances in individual tests, L – falling distance averaged over all tests in experimental sub-set.
